# BUB1 in cancer genetics and oncogenomics: from chromosomal instability to therapeutic vulnerabilities

**DOI:** 10.3389/fgene.2026.1832358

**Published:** 2026-06-09

**Authors:** Shuai Wang, Shizhen Liu, Yanan Zhou, Hongxiang Wang, Juxiang Chen

**Affiliations:** 1 Department of Neurosurgery, Shanghai Changhai Hospital, Naval Medical University, Shanghai, China; 2 Tongji University School of Medicine, Shanghai, China

**Keywords:** BUB1, chromosomal instability, immune evasion, oncogenomics, spindle assembly checkpoint, therapy resistance

## Abstract

BUB1 is a serine/threonine kinase and a core component of the spindle assembly checkpoint that safeguards faithful chromosome segregation during mitosis. Accumulating studies have shown that BUB1 is aberrantly expressed across multiple malignancies and contributes to tumor progression through both canonical mitotic functions and noncanonical extramitotic activities. In this review, we summarize the genetic and functional roles of BUB1 in cancer, with a focus on its involvement in chromosomal instability, aneuploidy, therapeutic resistance, and immune evasion. We discuss how BUB1 dysregulation is associated with adverse clinicopathological features and poor prognosis in several tumor types, while also highlighting context-dependent heterogeneity across cancers. Mechanistically, BUB1 contributes to malignant phenotypes by modulating mitotic checkpoint signaling, DNA damage responses, epithelial–mesenchymal transition, ferroptosis sensitivity, cancer stemness, and innate immune signaling. We further review current preclinical evidence supporting BUB1 as a therapeutic target, including the antitumor activity of small-molecule inhibitors and their synergistic potential in combination with radiotherapy, platinum agents, taxanes, and PARP inhibitors. Finally, we outline key unresolved questions, including the critical and still unresolved distinction between kinase-dependent and scaffold-dependent functions—which directly impacts inhibitor design and druggability—toxicity concerns in normal proliferating tissues, and the need for biomarker-guided patient stratification. Collectively, BUB1 represents a preclinically supported but still incompletely validated target in cancer genetics and oncogenomics.

## 1 Introduction

BUB1 (budding uninhibited by benzimidazoles 1) is a highly conserved serine/threonine kinase that functions as a central regulator of mitosis and a core component of the spindle assembly checkpoint (SAC). During cell division, BUB1 integrates catalytic and scaffold-dependent activities to ensure faithful chromosome segregation. Through phosphorylation of histone H2A at threonine 120, BUB1 promotes centromeric recruitment of shugoshin proteins, thereby preserving sister chromatid cohesion. In parallel, BUB1 serves as a kinetochore platform for the hierarchical assembly of checkpoint proteins, including BUB3 and the MAD1–MAD2 complex, ultimately facilitating mitotic checkpoint complex formation and restraining premature activation of the anaphase-promoting complex/cyclosome. This precise coupling between mechanical attachment status and checkpoint signaling is essential for maintaining chromosomal stability during mitosis. Dysregulation of BUB1, whether through insufficiency, mutation, or aberrant overexpression, can disrupt this fidelity and promote chromosomal instability (CIN) (Tucker et al., 2023), a hallmark of tumor evolution (Carter et al., 2006; Gordon et al., 2012).

Beyond its canonical role in mitotic surveillance, BUB1 has increasingly been implicated in cancer progression. Accumulating studies have shown that BUB1 is aberrantly upregulated in multiple aggressive malignancies, including pancreatic cancer, triple-negative breast cancer, lung cancer, glioblastoma, liver cancer, and sarcoma, where elevated expression is often associated with advanced stage (Piao et al., 2019; Huang et al., 2021), metastasis (Wang et al., 2024), treatment resistance (Cicirò et al., 2024), and poor survival (Long et al., 2021; Zhang et al., 2024). Importantly, the functional relevance of BUB1 in cancer appears to extend beyond its nuclear role in chromosome segregation. Emerging evidence suggests that BUB1 may modulate multiple cancer-associated processes, including PI3K/Akt and EGFR signaling, epithelial–mesenchymal transition, DNA damage repair (Wu et al., 2025), ferroptosis escape (Gao and Zou, 2026), and maintenance of cancer stem-like properties (Liu et al., 2026). More recently, a noncanonical cytoplasmic function of BUB1 has been described in the context of genotoxic stress, in which BUB1 restrains dsRNA accumulation and innate immune sensing, thereby contributing to immune evasion after radiotherapy. These findings collectively position BUB1 at the intersection of mitotic control, oncogenic signaling, therapeutic resistance, and tumor–immune interactions.

At the translational level, BUB1 has emerged as a potential preclinical therapeutic target. Small-molecule BUB1 inhibitors such as BAY-1816032 and related compounds have shown encouraging antitumor activity in preclinical models, either as single agents (Ricke et al., 2012) or in combination with taxanes (Weaver, 2014), platinum compounds, radiotherapy, and PARP inhibitors (Baron et al., 2016). Nevertheless, several major challenges remain unresolved, including incomplete understanding of BUB1 functions in normal proliferating tissues, uncertain therapeutic windows, the absence of clinical safety data, and the lack of robust biomarkers for patient selection. In addition, the clinical significance of BUB1 appears to be heterogeneous across tumor types, raising the possibility that its biological role may be context dependent rather than uniformly oncogenic. In this review, we summarize the canonical and noncanonical roles of BUB1 in cancer, with a particular focus on chromosomal instability, oncogenic signaling, immune escape, and therapeutic resistance. We also discuss the translational opportunities and major unresolved questions that must be addressed before BUB1-targeted strategies can be advanced toward clinical application.

Throughout this review, we distinguish between three levels of evidence when discussing the cancer relevance of BUB1: functional dependency, in which loss-of-function experiments demonstrate a causal contribution of BUB1 to tumor maintenance or therapy resistance; prognostic biomarker association, in which BUB1 expression level correlates with clinical outcome without established causality; and proliferation-associated expression, in which BUB1 upregulation may reflect increased mitotic activity in rapidly dividing tumors rather than a direct tumor-driving role. This distinction is essential for the accurate interpretation of the heterogeneous evidence base reviewed herein.

## 2 Canonical roles of BUB1 in mitosis and chromosomal stability

### 2.1 Structural features and kinetochore recruitment of BUB1

BUB1 is a multidomain serine/threonine kinase whose structural organization underlies its central role in mitotic control. It contains an N-terminal regulatory region, a central GLEBS motif, and a C-terminal kinase domain, enabling BUB1 to function not only as a catalytic enzyme (Bolanos-Garcia and Blundell, 2011) but also as a scaffold for checkpoint assembly (Williams et al., 2007; Goto et al., 2011). This dual catalytic and structural nature is essential for the coordination of chromosome segregation and maintenance of chromosomal stability.

The N-terminal region of BUB1 contains a conserved tetratricopeptide repeat (TPR) domain that mediates kinetochore targeting through interaction with centromere-associated proteins such as blinkin/KNL1. Beyond anchoring BUB1 at kinetochores, this region also contributes to the recruitment and spatial organization of key spindle checkpoint proteins, including BUBR1 and the MAD1–MAD2 complex. In the central region, the conserved GLEBS motif is required for BUB3 binding, thereby stabilizing kinetochore localization and reinforcing spindle assembly checkpoint signaling.

At the C terminus, BUB1 harbors a catalytically active kinase domain that phosphorylates histone H2A at Thr120, promoting shugoshin recruitment and supporting centromeric cohesion. Although its kinase activity is not essential for every aspect of spindle checkpoint activation, it is particularly important for chromosome alignment, centromeric signaling, and kinetochore–microtubule attachment stability. Thus, BUB1 acts as a molecular integrator that links structural kinetochore assembly with biochemical checkpoint output, and disruption of these functions may compromise mitotic fidelity and promote chromosomal instability.

Importantly, the catalytic and scaffold functions of BUB1 are mechanistically separable. Kinase activity is required for H2A-T120 phosphorylation and centromeric shugoshin recruitment, whereas the scaffold function—mediated primarily through the N-terminal TPR domain and central GLEBS motif—is essential for MAD1 kinetochore localization and mitotic checkpoint complex assembly. This functional duality has direct implications for therapeutic targeting: current small-molecule inhibitors such as BAY-1816032 selectively block kinase activity but do not disrupt scaffold-dependent checkpoint functions (Zhang et al., 2025a). Whether kinase inhibition alone is sufficient to abrogate the oncogenic potential of BUB1, or whether scaffold-directed strategies such as targeted protein degradation will be required, remains a key unresolved question that is further discussed in Sections 5 and 6.

### 2.2 BUB1 in spindle assembly checkpoint signaling

BUB1 is a central amplifier (Faesen et al., 2017) of spindle assembly checkpoint (SAC) signaling (Vanoosthuyse et al., 2004; Klebig et al., 2009). When kinetochores remain unattached or fail to establish proper tension, the kinase Mps1 phosphorylates multiple MELT repeats within KNL1, thereby creating dynamic docking sites for the BUB1–BUB3 complex. Once recruited to kinetochores, BUB1 not only contributes to the localization of BUBR1, but also promotes the spatial positioning of the MAD1–MAD2 complex in close proximity to KNL1, a key step required for efficient checkpoint signaling (London and Biggins, 2014).

This local enrichment of checkpoint components catalyzes the conformational conversion of soluble open MAD2 (O-MAD2) into its active closed form (C-MAD2). Newly generated C-MAD2 then associates with BUBR1, BUB3, and CDC20 to assemble the mitotic checkpoint complex (MCC), the major effector of SAC-mediated cell-cycle arrest. By sequestering CDC20 and functionally inhibiting the ubiquitin ligase activity of the anaphase-promoting complex/cyclosome (APC/C), the MCC prevents premature anaphase onset and ensures that chromosome segregation does not proceed until correct bipolar attachment has been achieved (Overlack et al., 2015).

In this framework, BUB1 serves as more than a passive checkpoint component. It acts as a spatial and signaling hub that couples kinetochore attachment status to downstream biochemical inhibition of APC/C. This function is particularly important for maintaining mitotic fidelity, because even subtle perturbation of BUB1-dependent SAC signaling may weaken checkpoint strength, permit chromosome missegregation, and create a permissive state for chromosomal instability. Thus, the role of BUB1 in SAC signaling provides a direct mechanistic bridge between defective mitotic surveillance and tumor-associated genome instability.

### 2.3 BUB1 dysregulation and chromosomal instability

Chromosomal instability (CIN) is one of the most important mechanistic links (Kops et al., 2005) between BUB1 dysfunction and tumorigenesis (Jeganathan et al., 2007). Notably, BUB1 dysregulation may promote CIN through two convergent routes: weakened checkpoint function caused by reduced BUB1 activity, and mitotic imbalance caused by pathological BUB1 overexpression. This bidirectional model highlights the central role of BUB1 in maintaining chromosome segregation fidelity.

When BUB1 expression is insufficient, or when germline alterations impair its checkpoint activity, spindle assembly checkpoint surveillance is weakened, allowing cells to enter anaphase before all chromosomes have achieved proper kinetochore–microtubule attachment. This increases chromosome missegregation and stochastic chromosome loss, and in experimental models has been linked to loss of heterozygosity involving key tumor suppressors such as p53 and Apc, thereby promoting tumor initiation and progression (Baker et al., 2009). These findings indicate that BUB1 insufficiency is not merely a marker of mitotic dysfunction, but can directly contribute to genome instability.

**FIGURE 1:**
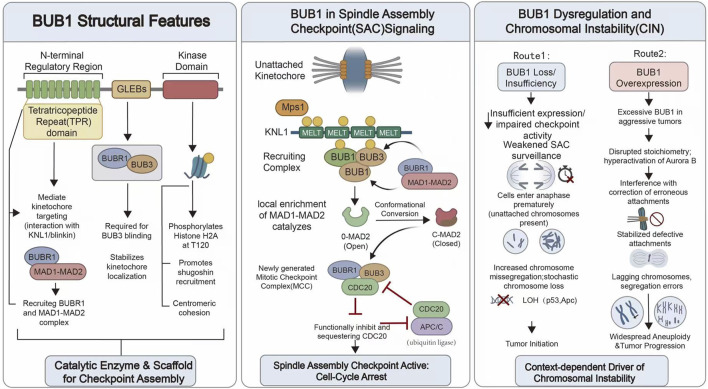
Structural and canonical mitotic functions of BUB1 in chromosome segregation and chromosomal instability. BUB1 is a multidomain serine/threonine kinase composed of an N-terminal regulatory region containing a tetratricopeptide repeat (TPR) domain, a central GLEBS motif required for BUB3 binding, and a C-terminal kinase domain. At kinetochores, BUB1 is recruited through Mps1-dependent phosphorylation of KNL1 MELT repeats and serves as both a scaffold and a catalytic regulator of spindle assembly checkpoint (SAC) signaling. BUB1 facilitates the recruitment of BUBR1 and the MAD1–MAD2 complex, promotes mitotic checkpoint complex (MCC) formation, and suppresses APC/C activation until proper bipolar attachment is achieved. In parallel, BUB1 phosphorylates histone H2A at Thr120, promoting shugoshin recruitment and centromeric cohesion. Dysregulation of BUB1 may promote chromosomal instability through two distinct routes: checkpoint insufficiency caused by reduced BUB1 activity, and mitotic imbalance caused by pathological BUB1 overexpression, which may induce Aurora B hyperactivation, defective kinetochore–microtubule attachment correction, chromosome missegregation, and aneuploidy.

Conversely, BUB1 overexpression can also drive CIN, particularly in aggressive tumors. Excessive BUB1 disrupts the stoichiometric balance of checkpoint and centromeric regulators and induces aberrant hyperactivation of Aurora B, which interferes with the correction of erroneous kinetochore–microtubule attachments. As a result, defective attachments may be stabilized, leading to lagging chromosomes, segregation errors, and widespread aneuploidy (Ricke et al., 2011). These observations suggest that both BUB1 deficiency and pathological overexpression can undermine mitotic accuracy through distinct mechanisms, supporting the view that BUB1 acts as a context-dependent regulator of chromosomal instability rather than a linear oncogenic factor with a single mode of action (Figure 1).

Critically, CIN generated by BUB1 dysregulation may serve as a unifying upstream mechanism linking many of the downstream phenotypes discussed in subsequent sections of this review. CIN-driven genomic heterogeneity provides the substrate for clonal selection under therapeutic pressure, while ongoing chromosome missegregation can generate cytosolic DNA and aberrant RNA species that engage innate immune sensing pathways. In this framework, the diverse extramitotic functions attributed to BUB1—including DNA damage tolerance (Section 4.2), ferroptosis resistance (Section 4.3), and immune evasion (Section 4.4)—can be understood not as independent oncogenic activities, but as convergent adaptive consequences of BUB1-mediated chromosomal instability.

## 3 Genetic and genomic dysregulation of BUB1 across cancers

### 3.1 Expression patterns and clinicopathological significance

BUB1 is aberrantly upregulated in a broad range of human malignancies, including pancreatic cancer, triple-negative breast cancer, non-small cell lung cancer, hepatocellular carcinoma, esophageal squamous cell carcinoma (ESCC) (Li et al., 2025), glioblastoma, osteosarcoma, soft-tissue sarcoma, renal cell carcinoma, endometrial cancer, cervical cancer, and colorectal cancer (Table 1). In many of these tumor types, elevated BUB1 expression has been associated with advanced stage, metastasis, therapeutic resistance, and shorter overall survival. These findings support the view that BUB1 is not only a mitotic regulator but also a clinically relevant marker of aggressive tumor behavior.

**TABLE 1:** Genetic and clinical relevance of BUB1 across major tumor types. (Evidence strength categories: Functional, loss-of-function or inhibitor experiments demonstrating causal role; Correlative, expression–outcome association with limited functional validation; Bioinformatic, database-derived analysis without experimental validation; Genetic, germline mutation evidence).

Tumor type	Type of alteration/evidence	Main biological role	Clinical association	Key limitation	Representative references
Pancreatic ductal adenocarcinoma (PDAC)	**[Functional]**Overexpression in tumor tissue; functional and prognostic studies	Suppresses ferroptosis through the NF2/MOB1-YAP axis and promotes gemcitabine resistance	Associated with gemcitabine resistance and poor prognosis; may have prognostic value	Evidence is still mainly preclinical and based on limited tumor-specific cohorts	Piao et al. (2019), Li et al. (2022), Wang et al. (2024c)
Breast cancer (including TNBC)	**[Functional]** High expression; validated through clinical transcriptomic cohorts, *in vitro* functional assays, and xenograft models	Contributes to chromosomal instability (CIN) and therapy resistance; promotes tumor progression by actively maintaining cancer stem cell (CSC) properties and self-renewal	Significantly associated with aggressive clinicopathological features, poor overall survival, and tumor recurrence	The precise molecular crosstalk linking BUB1-associated stemness, CIN, and immune microenvironment remodeling remains incompletely characterized in fully immunocompetent systems	Takagi et al. (2013), Han et al. (2015), Chen et al. (2022), Sriramulu et al. (2024a), Sriramulu et al. (2024b)
Glioblastoma (GBM)	**[Functional]**Overexpression; mechanistic study in preclinical models	Cytoplasmic BUB1 phosphorylates PABPC1, suppresses dsRNA sensing, and promotes post-radiation immune evasion	High expression is associated with shorter overall survival	Strong mechanistic evidence is available, but tumor-type validation is still limited	Hu et al. (2025)
Small cell lung cancer (SCLC)	**[Correlative]**Upregulation in tumor models	Activates AKT/mTOR signaling and promotes EMT	Associated with more aggressive behavior and worse prognosis	Evidence is largely model-based and not yet broadly validated clinically	Wang et al. (2024b)
Non-small cell lung cancer (NSCLC)	**[Correlative]** High expression; expression profiling and functional association studies	Regulates mitotic checkpoint function and affects sensitivity to chemotherapy and radiotherapy	High expression is associated with poor prognosis and treatment resistance	Mechanistic validation of chemoradiotherapy sensitization relies entirely on preclinical models; prospective clinical data remains absent	Wang et al. (2020), Nyati et al. (2023), Thoidingjam et al. (2024)
Osteosarcoma (OS)	**[Functional]**Overexpression; functional inhibition studies	Activates PI3K/Akt and ERK pathways to promote proliferation, migration, and invasion	Associated with stage progression and metastasis	Mostly based on preclinical functional studies	Huang et al. (2021)
Multiple myeloma (MM)	**[Functional]**Overexpression in malignant cells	Promotes chromosome segregation errors and chromosomal instability	Associated with disease progression and treatment resistance	Evidence is disease-specific and mainly mechanistic rather than broadly clinical	Fujibayashi et al. (2020)
Gastric cancer (GC)	**[Functional]** High expression in tumor tissue; mechanistic and prognostic studies	Activates the TRAF6/NF-κB/FGF18 axis through m6A-related regulation and promotes proliferation/metastasis	Associated with metastasis risk and chemoresistance	Prognostic direction may be context dependent because another gastric cohort linked low BUB1 to worse outcome	Stahl et al. (2017), Wang et al. (2024a)
Oral squamous cell carcinoma (OSCC)	**[Bioinformatic]** High expression; prognostic and immune-correlation analysis	Associated with high TMB, increased immune checkpoint expression, and reduced CD56^dim^ NK-cell infiltration	May have prognostic and immunotherapy-response predictive value	Evidence is largely correlative and not yet mechanistically resolved	Li (2025)
Hepatocellular carcinoma (HCC)	**[Functional]** High expression; mechanistic and prognostic studies	Promotes proliferation *via* SMAD2 phosphorylation and accelerates cell-cycle progression	High expression is associated with poor prognosis	Functional evidence exists, but broader validation across cohorts remains limited	Zhu et al. (2020), Zhang et al. (2025b)
Anaplastic thyroid carcinoma (ATC)	**[Functional]**Upregulation; functional studies	BUB1/KIF14 complex promotes chromosomal instability, progression, and metastasis	Associated with distant metastasis and worse survival	Evidence is based on a relatively limited disease context	Jin et al. (2024)
Non-muscle-invasive bladder cancer (NMIBC)	**[Bioinformatic]** High expression; RNA-seq plus qPCR validation	Biomarker-type association with progression	Predicts tumor progression and may serve as a prognostic biomarker	Primarily biomarker evidence, with limited mechanistic support	Piao et al. (2021)
Early-onset colorectal cancer (CRC)	**[Genetic]** Germline mutation and susceptibility evidence	Germline BUB1/BUB3 defects promote mosaic aneuploidy and cancer predisposition	Mutation carriers show increased colorectal cancer risk	This row reflects inherited susceptibility rather than the common overexpression pattern seen in other solid tumors	de Voer et al. (2013)
Sarcoma	**[Bioinformatic]** High expression in combined expression analyses	High expression of BUB1/BUB1B/BUB3 is associated with aggressive phenotype	High BUB1 expression is associated with worse survival	Core evidence reflects combined family-member analysis rather than BUB1-only mechanistic proof	Long et al. (2021)
Esophageal squamous cell carcinoma (ESCC)	**[Functional]** High expression in tumor tissues and cells; functional validation (proliferation/migration) and clinicopathological correlation	Promotes proliferation, migration, and invasion	Significantly associated with poor tumor differentiation, lymph node metastasis, and advanced clinical stage	Functional mechanisms remain phenomenological; deeper molecular targets mediating these phenotypes are unexplored	Li et al. (2025)
Renal cell carcinoma (KIRC)	**[Functional]** High expression; bioinformatic analysis with functional validation (knockdown)	Promotes proliferation and EMT *via* PI3K/Akt pathway; associated with immune infiltration and immunotherapy response	High expression associated with poor overall survival and advanced stage	Functional validation is based on cell line models; clinical cohort validation remains limited	Zi et al. (2025)

It should be noted that the strength of evidence supporting a direct oncogenic role for BUB1 varies substantially across tumor types. In a subset of cancers—including TNBC (Han et al., 2015; Sriramulu et al., 2024a; Sriramulu et al., 2024b), GBM (Hu et al., 2025), and PDAC (Wang et al., 2024c)—functional dependency has been demonstrated through loss-of-function experiments showing that BUB1 depletion impairs tumor cell viability, therapy resistance, or immune evasion. In contrast, for many other tumor types listed in Table 1, the available evidence remains primarily correlative, based on expression–outcome associations derived from transcriptomic profiling. Whether BUB1 overexpression in these settings reflects a genuine tumor-promoting dependency or instead accompanies a broader mitotic and proliferative gene program remains an important unresolved question. Moreover, the prognostic value of BUB1 may further depend on molecular subtype and treatment context, as the available evidence ranges from protein-level validation in some cancers to purely transcriptomic inference in others.

Overall, the available evidence suggests that BUB1 overexpression is frequently linked to malignant progression, but its role is likely context dependent rather than universally oncogenic. Therefore, BUB1 is better viewed as a candidate biomarker whose clinical relevance requires tumor-specific interpretation and further validation in large, well-annotated cohorts.

### 3.2 Germline mutations and genomic alterations

In addition to aberrant overexpression, inherited and genomic alterations of BUB1 further support its relevance in cancer genetics. Germline mutations affecting spindle checkpoint components, including BUB1, have been reported in patients with colorectal cancer predisposition and are associated with mosaic variegated aneuploidy, highlighting the importance of intact BUB1 function in safeguarding chromosome segregation fidelity (de Voer et al., 2013). These findings indicate that BUB1 is not only involved in tumor progression at the expression level, but may also contribute to inherited cancer susceptibility through disruption of mitotic checkpoint integrity.

At the somatic level, cancer-associated BUB1 dysregulation is more commonly manifested as transcriptional upregulation or functional imbalance rather than recurrent hotspot mutation. This pattern suggests that, in most tumors, BUB1 may act less as a classically mutated driver gene and more as a mitotic regulator whose abnormal dosage contributes to chromosomal instability, aneuploidy, and tumor evolution. Accordingly, the biological consequences of BUB1 alteration should be interpreted in the broader context of checkpoint dysfunction, genomic instability, and tumor-specific dependency rather than mutation status alone.

Overall, current evidence supports a dual view of BUB1 genomic dysregulation: rare germline defects may increase cancer susceptibility by weakening checkpoint fidelity, whereas more common expression-level abnormalities may contribute to malignant progression by sustaining chromosomal instability. This distinction is important for both mechanistic interpretation and future biomarker development.

### 3.3 Context-dependent prognostic heterogeneity

Although elevated BUB1 expression is frequently associated with poor clinical outcome, its prognostic value is not uniform across tumor types. In several malignancies—including pancreatic cancer (Piao et al., 2019), breast cancer (Takagi et al., 2013), liver cancer (Zhang et al., 2025b), neuroblastoma (Song et al., 2022), and sarcoma (Long et al., 2021)—high BUB1 expression has been linked to aggressive clinicopathological features and shortened survival, supporting its relevance as a marker of adverse disease biology. However, the strength of this association varies according to tumor lineage, molecular subtype, and the type of evidence available, ranging from transcriptomic inference to protein-level or functional validation.

Of note, the prognostic performance of BUB1 may be improved when it is incorporated into multigene risk models rather than assessed as a single marker. In prostate cancer, a four-gene signature including BUB1 was constructed using LASSO and Cox regression and externally validated in GEO cohorts; patients classified as high risk exhibited features associated with metastatic progression and differential predicted sensitivity to immunotherapeutic agents (Luo et al., 2025). Although this composite approach outperformed single-gene analysis in discriminating risk groups within the training and validation datasets, it has not yet been tested prospectively, and the added predictive value of BUB1 relative to other signature components remains unclear. These results suggest that integrating BUB1 into broader transcriptomic panels may enhance risk stratification, but independent clinical validation is required before such models can inform treatment decisions.

Importantly, BUB1 does not appear to behave as a uniformly unfavorable biomarker in every cancer context. A notable exception is gastric adenocarcinoma, in which low BUB1 expression has been reported as an adverse prognostic marker (Stahl et al., 2017). This finding suggests that the biological significance of BUB1 may depend on the proliferative state, checkpoint dependency, and compensatory signaling landscape of a given tumor rather than on expression level alone. In this regard, BUB1 may function as a context-dependent biomarker whose clinical interpretation requires tumor-specific rather than pan-cancer generalization.

Current evidence supports a nuanced view of BUB1 prognosis biology: high expression often accompanies aggressive disease, but the direction and magnitude of prognostic association are not invariant across cancers. Therefore, future studies should combine expression data with molecular subtype, chromosomal instability status, and treatment context to define when BUB1 is most informative as a prognostic marker and when its apparent association may reflect broader mitotic or proliferative programs rather than a direct tumor-driving role.

### 3.4 Evidence gaps and underexplored tumor types

Although BUB1 overexpression has been well documented in several tumor types with functional validation—most notably TNBC, GBM, and PDAC—the evidence base remains uneven across cancers. Several clinically important malignancies have received comparatively limited attention in the BUB1 literature, and the available evidence in these settings warrants careful interpretation. In endometrial cancer, bioinformatic analyses have identified BUB1 as a gene significantly correlated with tumor development and prognosis, with high expression associated with adverse clinical features (Zhang et al., 2024). However, these findings are derived primarily from transcriptomic profiling without independent functional validation, and whether BUB1 contributes mechanistically to endometrial tumorigenesis or simply reflects a proliferative gene signature remains to be determined. In cervical cancer, BUB1 has been implicated in ferroptosis resistance through an indirect regulatory axis involving ALYREF-mediated m5C methylation of KIF20A, which activates the KIF20A/BUB1 pathway under hypoxic conditions (Gao and Zou, 2026). While this study provides an initial mechanistic link between BUB1 and metabolic adaptation in cervical cancer, the evidence is limited to a single *in vitro* model, and broader validation across cervical cancer subtypes and clinical cohorts is needed. In colorectal cancer, BUB1 has been identified as a molecular target of the natural compound nitidine chloride, with evidence from molecular dynamics simulation, spatial transcriptomics, and single-cell RNA sequencing supporting a role for BUB1 in colorectal tumor cell proliferation (Wu et al., 2025). In addition, germline mutations in BUB1 have been reported as risk factors for early-onset colorectal cancer predisposition (de Voer et al., 2013). Together, these findings suggest that BUB1 may contribute to colorectal cancer through both somatic overexpression and inherited checkpoint deficiency, although functional dependency has not been established through conventional loss-of function experiments.

In renal cell carcinoma, a recent study demonstrated that BUB1 is overexpressed in kidney renal clear cell carcinoma (KIRC) tissues and cell lines, and that BUB1 knockdown suppresses cell proliferation, migration, and epithelial–mesenchymal transition through inactivation of the PI3K/Akt pathway (Zi et al., 2025). This study provides one of the few examples of functional validation of BUB1 dependency in renal cancer, although broader cohort-level and *in vivo* validation remains limited. In mesothelioma, direct BUB1-specific evidence has recently begun to emerge, although the tumor-specific literature remains limited. Earlier transcriptomic studies of malignant pleural mesothelioma (MPM) indicated significant deregulation of the spindle checkpoint pathway, supporting the biological plausibility of mitotic checkpoint vulnerabilities in this disease. More recently, a genome-wide CRISPR screen and validation study identified BUB1 kinase as a druggable vulnerability in MPM, showing that BUB1 is elevated in tumor tissues, that higher BUB1 expression is associated with shorter patient survival, and that genetic depletion or pharmacologic inhibition of BUB1 suppresses tumor cell growth while inducing G2/M arrest, senescence, and apoptosis. These findings suggest that BUB1 may represent a biologically meaningful dependency and a potential therapeutic target in mesothelioma. However, the current evidence remains limited in scope, and broader cohort-level, *in vivo*, and mechanistic validation will be required before mesothelioma can be considered a well-established BUB1-dependent tumor context (Cakiroglu et al., 2025). These evidence gaps highlight a broader challenge in the BUB1 field: for many tumor types, the available data remain correlative or bioinformatic, making it difficult to distinguish genuine functional dependency from proliferation-associated expression. Future studies should prioritize loss-of-function experiments and pharmacologic validation in tumor types where BUB1 overexpression has been observed but not yet functionally interrogated. Establishing a clearer hierarchy of evidence across cancers will be essential for identifying the tumor contexts in which BUB1 is most likely to represent a therapeutically relevant target.

## 4 Oncogenic functions of BUB1 beyond canonical mitosis

### 4.1 Proliferation and epithelial–mesenchymal transition

Beyond its canonical role in mitotic checkpoint control, BUB1 has been implicated in the regulation of tumor cell proliferation (Grabsch et al., 2003) and epithelial–mesenchymal transition (EMT) (Zhang et al., 2025a). In several tumor models, elevated BUB1 expression is associated with enhanced proliferative capacity, increased migratory potential, and more aggressive biological behavior, suggesting that its oncogenic relevance extends beyond chromosome segregation alone.

Recent work has identified epitranscriptomic regulation as one upstream mechanism controlling BUB1 abundance. In hepatocellular carcinoma, Zhang et al. reported that the m6A demethylase FTO is overexpressed in tumor tissue and stabilizes BUB1 mRNA through a YTHDF2-dependent pathway (Zhang et al., 2025a). Functionally, accumulated BUB1 protein was shown to physically interact with TGF-βR1 by co-immunoprecipitation, and FTO knockdown attenuated TGF-β downstream signaling, reduced EMT marker expression, and suppressed tumor growth in xenograft models (Zhang et al., 2025a). These findings link RNA methylation status to BUB1 protein levels and suggest that BUB1 may serve as a downstream effector connecting epigenetic dysregulation to pro-metastatic signaling in HCC. However, this mechanism has so far been characterized in a single tumor type, and whether similar epitranscriptomic control of BUB1 operates in other cancers remains to be determined.

Mechanistically, BUB1 appears to promote proliferation through multiple context-dependent signaling pathways. In hepatocellular carcinoma, BUB1 has been reported to enhance tumor cell proliferation by activating SMAD2 phosphorylation (Zhu et al., 2020). In gastric cancer, BUB1 potentiates proliferation and metastasis through the TRAF6/NF-κB/FGF18 axis (Wang et al., 2024a). In addition, studies in NSCLC and breast cancer indicate that BUB1 can regulate EGFR signaling by reducing receptor internalization, thereby sustaining downstream proliferative signaling (Nyati et al., 2023).

**FIGURE 2:**
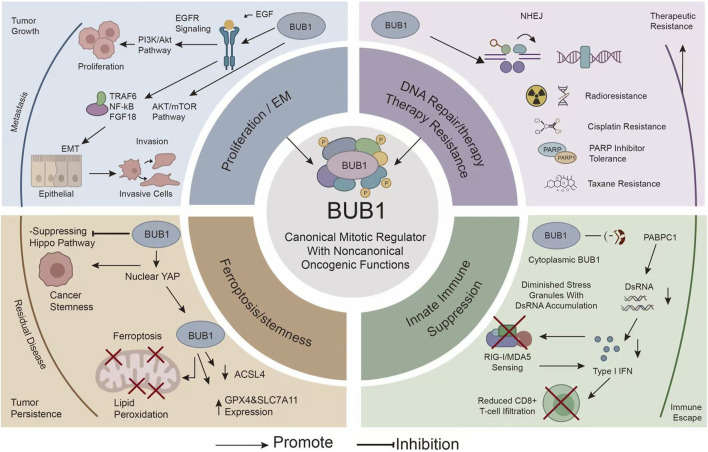
Noncanonical oncogenic functions of BUB1 in cancer progression. Beyond its canonical role in mitotic checkpoint control, BUB1 contributes to tumor progression through multiple noncanonical mechanisms. BUB1 promotes proliferation and epithelial–mesenchymal transition (EMT) by modulating signaling pathways such as PI3K/Akt, AKT/mTOR, EGFR, and TRAF6/NF-κB/FGF18 in a tumor-context-dependent manner. BUB1 also supports DNA damage repair and therapeutic resistance, including NHEJ-dependent radioresistance and reduced sensitivity to chemotherapy and PARP inhibition. In addition, BUB1 suppresses ferroptosis and maintains cancer stem-like properties by regulating pathways associated with NF2/MOB1–YAP and ferroptosis-related effectors such as GPX4, SLC7A11, and ACSL4. Under genotoxic stress, cytoplasmic BUB1 may promote immune evasion by phosphorylating PABPC1, thereby limiting dsRNA accumulation, suppressing RIG-I/MDA5-mediated innate immune sensing, and reducing type I interferon signaling and T-cell infiltration. These pathways collectively link BUB1 to tumor growth, metastasis, therapy resistance, and immune escape.

BUB1 has also been linked to EMT-associated phenotypes through several of the same signaling pathways implicated in BUB1-dependent therapy resistance (discussed in Section 4.2). In small cell lung cancer, BUB1 activates AKT/mTOR signaling and has been linked to EMT (Wang et al., 2024b), while in osteosarcoma, BUB1 inhibition suppresses PI3K/Akt and ERK pathway activity (Huang et al., 2021). The overlap between these proliferative and pro-migratory pathways and the DNA damage tolerance mechanisms described below suggests convergent signaling rather than independent oncogenic programs. However, direct evidence linking BUB1 to core EMT transcriptional regulators (such as SNAI1, ZEB1, or TWIST) remains limited, and the contribution of BUB1 to EMT should be considered context dependent (Figure 2).

### 4.2 DNA damage repair and therapy resistance

In addition to promoting tumor growth, BUB1 contributes to therapeutic resistance by supporting DNA damage repair and preserving survival under genotoxic stress. Recent studies suggest that BUB1 inhibition can impair both homologous recombination (HR) and non-homologous end joining (NHEJ), thereby lowering the tolerance of tumor cells to DNA-damaging therapies and creating opportunities for combination treatment (Siemeister et al., 2019; Sriramulu et al., 2024b).

A more direct mechanistic link has been established in triple-negative breast cancer, where BUB1 regulates the NHEJ pathway and mediates radioresistance (Sriramulu et al., 2024a). Consistent with this, pharmacologic inhibition of BUB1 with BAY-1816032 sensitizes TNBC cells to chemotherapy and radiotherapy, and shows synergy with cisplatin, taxanes, and PARP inhibitors in preclinical models (Siemeister et al., 2019; Sriramulu et al., 2024b). These findings suggest that BUB1 supports therapy resistance not only through mitotic adaptation but also through active maintenance of DNA repair capacity.

BUB1-dependent resistance may also extend beyond breast cancer. In prostate cancer models, inhibition of BUB1 has been reported to reverse taxane resistance, restoring sensitivity to microtubule-stabilizing agents even in resistant settings (Martinez et al., 2023). Together, these data support a model in which BUB1 functions as a context-dependent resistance factor that links checkpoint signaling, DNA repair proficiency, and treatment adaptation. However, most available evidence remains preclinical, and the extent to which these mechanisms are conserved across tumor types requires further validation.

### 4.3 Ferroptosis escape and cancer stemness

Beyond canonical mitotic functions, BUB1 may also promote tumor adaptation to metabolic stress (Hanahan and Weinberg, 2011) by suppressing ferroptosis (Dixon et al., 2012; Hassannia et al., 2019) and sustaining cancer stem-like properties (Sha et al., 2021). In pancreatic ductal adenocarcinoma, BUB1 overexpression has been reported to inhibit the NF2/MOB1–YAP signaling axis, thereby reducing sensitivity to lipid peroxidation and promoting gemcitabine resistance (Wang et al., 2024c). This finding suggests that BUB1 can support tumor survival not only through cell-cycle control, but also through metabolic protection under therapeutic stress.

Evidence from lung adenocarcinoma suggests that BUB1-mediated ferroptosis suppression is not confined to pancreatic and breast cancer. Mo et al. reported that BUB1 promotes LUAD progression by activating STAT3 signaling, which in turn sustains GPX4 expression and limits lipid peroxidation (Mo et al., 2025). This mechanism is distinct from the NF2/MOB1–YAP axis identified in PDAC (Wang et al., 2024c), indicating that BUB1 engages different downstream effectors to achieve anti-ferroptotic protection depending on tumor lineage. The identification of at least two independent anti-ferroptotic pathways downstream of BUB1 raises a practical question for therapeutic development: whether BUB1 kinase inhibition alone is sufficient to override these parallel survival programs, or whether pathway-specific co-targeting will be required to fully restore ferroptosis sensitivity across tumor types. At present, this question remains unanswered, and further comparative studies using matched BUB1 inhibition across multiple tumor models are needed. In parallel, blockade of BUB1 has been shown to impair cancer stem-like potential in breast cancer models, indicating that BUB1-dependent reprogramming may also contribute to self-renewal and residual disease persistence (Han et al., 2015).

These observations support a model in which BUB1 enhances tumor fitness by coupling ferroptosis resistance to stem-like maintenance. However, current evidence remains limited to selected tumor models, and the generalizability of this mechanism across cancers requires further validation.

### 4.4 Innate immune suppression and immune evasion

Beyond its canonical mitotic functions, BUB1 has recently been implicated in tumor immune evasion through a noncanonical cytoplasmic mechanism. Under genotoxic stress, including radiotherapy, BUB1 undergoes nucleo-cytoplasmic redistribution and phosphorylates the RNA-binding protein PABPC1, thereby promoting its ubiquitin-dependent degradation. Loss of PABPC1 prevents the accumulation of endogenous dsRNA within stress granules, leading to suppression of RIG-I/MDA5-mediated innate immune sensing and reduced type I interferon signaling (Hu et al., 2025).

Functionally, this pathway limits antitumor immune activation after DNA damage. In preclinical glioblastoma models, inhibition of BUB1 restores dsRNA sensing, reactivates interferon signaling, and enhances intratumoral CD8^+^ and CD4^+^ T-cell infiltration, supporting a role for BUB1 in therapy-induced immune escape (Hu et al., 2025). These findings suggest that BUB1 can suppress antitumor immunity not merely as a consequence of rapid proliferation, but through an active and targetable mechanism of innate immune restraint.

Correlative evidence further supports an immunomodulatory role for BUB1. In oral squamous cell carcinoma, high BUB1 expression has been associated with increased tumor mutational burden and elevated expression of multiple immune checkpoint molecules, but also with reduced CD56^dim^ natural killer-cell infiltration, indicating a potentially immune-suppressed or immune-excluded phenotype (Li, 2025). Consistent with these observations, a recent transcriptomic analysis in breast cancer reported an inverse correlation between BUB1 expression and CD8^+^ T-cell infiltration, and further suggested that high BUB1 expression may be associated with reduced responsiveness to immune checkpoint blockade (Zhou et al., 2024). While these findings raise the possibility that BUB1 status could inform immunotherapy patient selection, the evidence remains correlative and is derived from retrospective cohort analysis without prospective validation. However, these association-based observations should be interpreted cautiously, and broader functional validation across additional tumor types is needed to establish whether BUB1 directly modulates the tumor immune microenvironment or instead reflects a proliferative gene program that confounds immune infiltration estimates.

## 5 Therapeutic targeting of BUB1

### 5.1 Preclinical inhibitors and pharmacological effects

**FIGURE 3:**
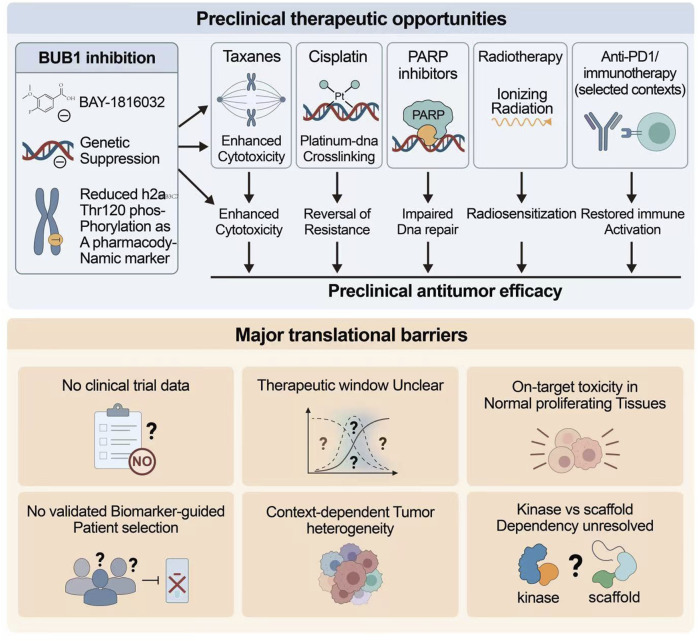
Preclinical therapeutic strategies and translational challenges of BUB1 targeting. Current therapeutic targeting of BUB1 remains preclinical but has shown antitumor activity in multiple preclinical tumor models. Small-molecule inhibition, particularly with BAY-1816032, suppresses BUB1 kinase activity and inhibits H2A Thr120 phosphorylation, providing a pharmacodynamic readout of target engagement. In preclinical studies, BUB1 inhibition enhances sensitivity to taxanes, cisplatin, PARP inhibitors, radiotherapy, and, in selected contexts, immunotherapy. Genetic or pharmacologic BUB1 suppression may also reverse ferroptosis escape, overcome taxane resistance, and restore innate immune activation after genotoxic stress. However, several major barriers limit clinical translation, including the absence of clinical trial data, uncertain therapeutic window, potential on-target toxicity in normal proliferating tissues, lack of validated biomarkers for patient selection, context-dependent tumor heterogeneity, and incomplete understanding of kinase-dependent *versus* scaffold-dependent vulnerabilities. These features position BUB1 as a preclinically supported but still incompletely validated target whose future development will likely depend on rational combination strategies and biomarker-guided stratification.

Pharmacologic targeting of BUB1 remains entirely at a preclinical stage, and no BUB1 inhibitor has entered clinical evaluation to date. Nevertheless, available preclinical studies provide initial evidence supporting the feasibility of BUB1 inhibition as an investigational antitumor approach. Among currently reported compounds, BAY-1816032 is the best-characterized small-molecule BUB1 inhibitor and has shown potent target engagement in tumor models. Mechanistically, BAY-1816032 suppresses BUB1 kinase activity and blocks phosphorylation of histone H2A at Thr120, a key downstream event required for centromeric signaling and chromosome segregation control (Siemeister et al., 2019). This on-target pharmacodynamic effect provides a practical experimental readout for BUB1 inhibition *in vitro* and *in vivo* (Figure 3).

In preclinical cancer models, BAY-1816032 exhibits direct antiproliferative activity as a single agent, with growth inhibition reported in triple-negative breast cancer and lung cancer cell lines at micromolar concentrations. In addition to reducing tumor cell viability, BUB1 inhibition disrupts mitotic progression and increases susceptibility to chromosome segregation defects, supporting the concept that BUB1-dependent checkpoint integrity constitutes a targetable vulnerability in rapidly proliferating tumors (Siemeister et al., 2019). By contrast, related compounds such as BAY-320 and BAY-1816031 appear to have more limited pharmacological performance, suggesting that inhibitor selectivity and potency remain important constraints in this field.

An additional consideration is the mechanistic scope of kinase-directed inhibition. As discussed in Section 2.1, BUB1 exerts both catalytic and scaffold-dependent functions, and current inhibitors such as BAY-1816032 selectively block kinase activity without disrupting scaffold-mediated checkpoint protein recruitment. This raises the question of whether kinase inhibition alone can fully recapitulate the antitumor effects observed with genetic BUB1 depletion, which ablates both functions simultaneously. Alternative modalities such as proteolysis-targeting chimeras (PROTACs) or molecular glue degraders could in principle eliminate both kinase and scaffold functions, but such approaches for BUB1 have not yet been reported.

Despite these encouraging findings, the current evidence base remains entirely preclinical. No BUB1 inhibitor has yet entered clinical evaluation, and key translational issues—including therapeutic window, on-target toxicity in normal proliferating tissues, and biomarker-guided patient selection—remain unresolved (Table 2). Therefore, available BUB1 inhibitors should currently be viewed as pharmacological tools and early translational leads rather than clinically validated agents. At present, the clinical readiness of BUB1-targeted therapy remains low, with the field still at the stage of preclinical proof-of-concept.

**TABLE 2:** Preclinical therapeutic strategies targeting BUB1.

Agent/intervention	Cancer model	Combination partner	Main phenotype	Evidence level	Translational limitation	Representative references
BAY-1816032	TNBC (SUM159, MDA-MB-231, HCC1937)	Olaparib, cisplatin, paclitaxel, radiotherapy	Enhances chemo/radiosensitivity, induces synergistic apoptosis, and sensitizes BRCA1/2 wild-type cells to PARP inhibition	Cell-based and preclinical pharmacologic evidence with quantitative synergy metrics	Evidence is confined to preclinical models; long-term safety and therapeutic window remain undefined	Siemeister et al. (2019), Sriramulu et al. (2024b)
BAY-1816032	NSCLC (A549, H1299) and SCLC models	Cisplatin, paclitaxel, radiotherapy	Suppresses proliferation, enhances chemo/radiotherapy-induced killing, and induces G2/M arrest	Cell-based pharmacologic evidence	Support is mainly preclinical and partly model-restricted; clinical relevance is not yet established	Siemeister et al. (2019), Thoidingjam et al. (2024)
BAY-320/BAY-1816031, or genetic BUB1 suppression	PDAC (PANC-1, Mia PaCa-2)	Gemcitabine	Reverses NF2/MOB1-YAP-associated ferroptosis escape, reduces tumor burden, and restores gemcitabine sensitivity	Cell and xenograft-level preclinical evidence	Attribution to individual inhibitors requires caution; some evidence is based on BUB1 knockdown rather than a single validated drug lead	Baron et al. (2016), Wang et al. (2024c)
siRNA/shRNA BUB1 knockdown	Glioblastoma (U251 orthotopic/xenograft models)	Radiotherapy, anti-PD1	Restores dsRNA sensing, activates RIG-I/MDA5 and type I IFN signaling, and enhances CD8+/CD4+ T-cell infiltration	Mechanistic *in vivo* preclinical evidence	Primarily genetic rather than drug-based evidence; broader tumor validation is still limited	Hu et al. (2025)
BAY-1816032	Prostate cancer (DU-145, PC-3; taxane-resistant models)	Docetaxel, cabazitaxel	Reverses AR-variant-driven taxane resistance and restores sensitivity to microtubule-stabilizing agents	Preclinical resistance-reversal evidence in cell models	No clinical validation; applicability beyond resistant prostate cancer remains uncertain	Martinez et al. (2023)
BUB1 shRNA knockdown	Breast cancer (including TNBC)	Monotherapy (Genetic target validation)	Impairs cancer stem cell (CSC) potential, significantly reduces mammosphere formation, and suppresses *in vivo* tumorigenicity	Genetic validation of BUB1 dependency for CSC maintenance and tumor self-renewal	Not directly translatable as a systemic therapeutic modality; strictly supports target validity rather than clinical drug readiness	Han et al. (2015)

Abbreviations: TNBC, triple-negative breast cancer; NSCLC, non-small cell lung cancer; SCLC, small cell lung cancer; PDAC, pancreatic ductal adenocarcinoma; IFN, interferon.

### 5.2 Combination strategies with chemotherapy, radiotherapy, and PARP inhibition

The translational value of BUB1 inhibition appears to be most pronounced in combination settings. Rather than serving only as a single-agent antiproliferative strategy, pharmacologic blockade of BUB1 can enhance the efficacy of multiple anticancer treatments by weakening mitotic fidelity and reducing DNA damage repair capacity. This creates a rationale for combining BUB1 inhibitors with therapies that impose replication stress, spindle stress, or genotoxic injury (Siemeister et al., 2019; Sriramulu et al., 2024b).

Preclinical studies have shown that BAY-1816032 sensitizes tumor cells to taxanes, cisplatin, and PARP inhibitors both *in vitro* and *in vivo* (Siemeister et al., 2019). In preclinical TNBC models, BUB1 inhibition also enhanced responsiveness to chemotherapy and radiotherapy, at least in part through suppression of non-homologous end joining (NHEJ) and impaired repair of treatment-induced DNA damage (Sriramulu et al., 2024a; Sriramulu et al., 2024b). These findings support a model in which BUB1 blockade amplifies therapeutic stress by coupling checkpoint disruption to defective damage tolerance.

Combination benefit may also extend to resistant disease states. In prostate cancer models, inhibition of BUB1 has been reported to reverse taxane resistance, suggesting that BUB1 may help sustain survival programs under chronic microtubule-directed therapy (Martinez et al., 2023). These data indicate that BUB1 inhibition is unlikely to be most useful as monotherapy, but may instead function as a sensitizing strategy in biomarker-defined tumors exposed to DNA-damaging or mitotic stress-inducing agents. However, this concept remains preclinical and requires validation in more diverse tumor models and clinically relevant treatment settings.

### 5.3 Translational opportunities and barriers

Current evidence positions BUB1 as a preclinically supported but still early-stage therapeutic target in cancer. Its translational appeal lies in three main features: its central role in mitotic fidelity, its ability to support DNA damage repair and therapy resistance, and its emerging noncanonical function in immune suppression under genotoxic stress. Together, these properties suggest that BUB1 inhibition may offer therapeutic benefit not only by impairing tumor cell division, but also by enhancing the efficacy of radiotherapy (Sriramulu et al., 2024a; Sriramulu et al., 2024b), chemotherapy (Siemeister et al., 2019; Sriramulu et al., 2024b), PARP inhibition (Siemeister et al., 2019), and potentially immunotherapy in selected settings (Hu et al., 2025).

However, several barriers currently limit clinical translation. First, all available BUB1-targeting strategies remain preclinical, and no inhibitor has yet entered clinical testing (Siemeister et al., 2019). Second, because BUB1 is also required for normal mitotic progression in proliferating tissues, the therapeutic window and risk of on-target toxicity remain uncertain. Third, there is still no validated biomarker framework to identify tumors most likely to benefit from BUB1 inhibition. Based on the available preclinical evidence, several tumor contexts may represent rational candidate populations for future investigation: (i) tumors with high chromosomal instability burden, where BUB1-dependent checkpoint maintenance may constitute a selective vulnerability; (ii) cancers with acquired resistance to taxanes or platinum agents, where BUB1 inhibition has shown preclinical resensitization activity (Siemeister et al., 2019; Zhang et al., 2025a); (iii) BRCA-wild-type tumors with intact homologous recombination, where BUB1 inhibition has been shown to enhance PARP inhibitor sensitivity in preclinical models, potentially by compromising DNA repair competence (Zhang et al., 2025a); and (iv) tumors treated with radiotherapy, particularly those with low baseline immune infiltration, where BUB1 blockade may simultaneously enhance DNA damage and restore innate immune sensing (Sriramulu et al., 2024a; Hu et al., 2025). However, all of these remain biologically plausible hypotheses rather than clinically validated indications, and prospective biomarker-guided trial designs will be essential.

An additional challenge is the context-dependent biology of BUB1 across tumor types. Although BUB1 overexpression is often associated with poor prognosis and aggressive behavior, this pattern is not universal; for example, low BUB1 expression has been linked to adverse outcome in gastric adenocarcinoma (Stahl et al., 2017). Such heterogeneity suggests that BUB1 should not be viewed as a uniformly actionable target across all cancers. Instead, future development will likely require tumor-specific interpretation, pharmacodynamic biomarkers of target engagement, and rational combination strategies tailored to defined molecular contexts (Stahl et al., 2017; Martinez et al., 2023).

BUB1 inhibition offers meaningful translational opportunities, particularly in combination-based strategies, but the field remains at the stage of mechanistic validation and preclinical optimization. Progress toward clinical application will depend on improved inhibitor selectivity, clearer toxicity assessment, and biomarker-guided trial design.

## 6 Discussion

### 6.1 Critical controversies and unresolved questions

The evidence reviewed in this manuscript supports a model in which chromosomal instability serves as the central mechanistic hub connecting BUB1 dysregulation to its diverse downstream phenotypes. BUB1 dysfunction—whether through insufficiency, overexpression, or stoichiometric imbalance—compromises chromosome segregation fidelity and generates ongoing genomic heterogeneity. This CIN-driven instability provides a unifying framework for understanding the extramitotic functions attributed to BUB1: CIN-induced replication stress and DNA damage create dependence on repair pathways that BUB1 helps sustain; genomic heterogeneity generated by CIN facilitates clonal adaptation to metabolic stress and ferroptosis; and aberrant nucleic acid species arising from chromosome missegregation engage innate immune pathways that BUB1 actively suppresses. Rather than viewing these as independent oncogenic activities, we propose that they represent convergent adaptive consequences of BUB1-mediated chromosomal instability. This interpretation is further supported by the observation that BUB1 overexpression has been most consistently associated with adverse prognosis in tumor types characterized by high levels of genomic instability, such as gliomas (Kops et al., 2005) and tumors with recurrent chromosomal gains and losses (Ricke et al., 2011), although a direct correlation between BUB1 expression and quantitative CIN metrics has not yet been systematically assessed across cancer types.

Although BUB1 is frequently overexpressed in aggressive tumors, its biological and clinical significance is highly context-dependent. In multiple malignancies—including breast carcinoma (Takagi et al., 2013), hepatocellular carcinoma (Zhang et al., 2025b), pancreatic ductal adenocarcinoma (Piao et al., 2019), and sarcoma (Long et al., 2021)—elevated BUB1 expression correlates with poor prognosis. However, the available evidence in these settings remains largely correlative, making it difficult to distinguish whether BUB1 acts as a true tumor-promoting dependency or instead reflects a highly proliferative mitotic state. This complexity is further underscored by the inverse finding in gastric adenocarcinoma, where low BUB1 expression paradoxically predicts an unfavorable clinical outcome (Stahl et al., 2017). Notably, published pan-cancer expression analyses have confirmed that BUB1 is broadly overexpressed across TCGA tumor types but that its prognostic impact varies substantially, with significant adverse associations in some cancers and no clear prognostic effect in others (Cicirò et al., 2024). This pattern suggests that BUB1 dependency may be restricted to tumors with specific molecular features rather than representing a universal oncogenic mechanism. Consequently, the prognostic and functional relevance of BUB1 is strongly context-dependent rather than universally oncogenic.

A second unresolved issue concerns the functional basis of BUB1 dependency in cancer. BUB1 exerts both kinase-dependent and scaffold-dependent activities—the latter being crucial for MAD1 kinetochore recruitment and SAC signaling (London and Biggins, 2014; Overlack et al., 2015). Yet, current therapeutic approaches primarily target its kinase domain. While kinase inhibition can successfully suppress H2A-T120 phosphorylation and centromeric signaling (Siemeister et al., 2019), it remains unclear whether this catalytic blockade is sufficient to neutralize the full spectrum of BUB1-driven tumor functions, particularly those relying on its structural scaffold properties. This question has direct translational implications: if scaffold-dependent functions prove to be the primary mediators of BUB1-driven tumor phenotypes in certain contexts, then kinase-directed small molecules may have limited single-agent efficacy, and alternative modalities such as targeted protein degradation would need to be prioritized in the drug development pipeline. Concurrently, the specific tumor subpopulations most likely to benefit from BUB1 inhibition remain poorly defined. Current preclinical evidence suggests candidate contexts should include cancers exhibiting acquired resistance to taxanes (Siemeister et al., 2019; Martinez et al., 2023), dependency on homologous recombination or non-homologous end joining for DNA repair (Sriramulu et al., 2024b), or specific resistance to radiotherapy (Sriramulu et al., 2024a). However, these remain biologically plausible hypotheses rather than clinically validated indications.

These mechanistic uncertainties are directly linked to major translational barriers. Crucially, all currently available BUB1-targeting strategies remain preclinical, and the therapeutic window is entirely undefined given the essential role of BUB1 in maintaining mitotic fidelity in normal proliferating tissues. Furthermore, no biomarker-guided framework has been established for patient stratification. The optimal integration of BUB1 inhibition into combination regimens requires tumor-specific validation: whether deployed alongside DNA-damaging chemotherapy (Sriramulu et al., 2024b), ionizing radiotherapy (Sriramulu et al., 2024a), PARP inhibitors to exploit replication stress (Siemeister et al., 2019), or emerging immunomodulatory strategies designed to reactivate dsRNA-mediated innate immune sensing (Hu et al., 2025). Current evidence establishes BUB1 as a compelling but incompletely validated target, whose clinical advancement strictly depends on enhanced pharmacological selectivity, rigorous toxicity profiling, and prospective biomarker-driven trial designs.

### 6.2 Conclusion and future perspectives

BUB1 is a central serine/threonine kinase in the spindle assembly checkpoint and plays an essential role in preserving chromosome segregation fidelity, regulating mitotic progression, and maintaining genomic stability. Across several tumor types, aberrant BUB1 expression has been associated with aggressive clinicopathological features, therapy resistance, and poor clinical outcomes, supporting its relevance as a potential prognostic biomarker and therapeutic target. Mechanistically, BUB1 contributes to tumor progression not only through its canonical functions in mitotic checkpoint signaling and chromosomal instability, but also through broader effects on oncogenic signaling, epithelial–mesenchymal transition, DNA repair, ferroptosis regulation, cancer stemness, and immune suppression. Published pan-cancer expression and survival analyses have further confirmed that BUB1 upregulation is widespread but heterogeneous across human cancers, with context-dependent prognostic significance that varies by tumor lineage and molecular subtype (Cicirò et al., 2024).

Current preclinical studies further suggest that BUB1 inhibition may represent an investigational therapeutic approach. Pharmacological or genetic suppression of BUB1 has shown antitumor activity in several cancer models and can enhance sensitivity to chemotherapy, radiotherapy, PARP inhibition, and immune checkpoint blockade in selected contexts. These findings support the concept that BUB1 may function as a therapeutically exploitable node linking chromosomal instability, DNA damage responses, and tumor immune remodeling. However, the available evidence remains largely preclinical, and several important limitations must be addressed before clinical translation can be considered. These include the absence of early-phase clinical safety data, the possibility of toxicity in normal proliferating tissues, the lack of prospective biomarker-guided stratification strategies based on CIN or aneuploidy-related features, and the marked heterogeneity of BUB1 biology across tumor types. Notably, the observation that low BUB1 expression is associated with unfavorable prognosis in certain gastric cancer cohorts highlights the need for a more nuanced and context-specific interpretation of BUB1 function rather than assuming a uniformly oncogenic role.

BUB1 should be viewed as a preclinically supported but still incompletely validated target in cancer genetics and oncogenomics. Future research should focus on clarifying the relative contribution of kinase-dependent *versus* scaffold-dependent functions, defining context-specific mechanisms across tumor types, improving pharmacological selectivity, and developing rational biomarker-guided combination strategies. Addressing these questions will be essential for determining whether BUB1 can ultimately be translated from a compelling experimental target into a clinically actionable therapeutic target.

## Generative Al statement

The author(s) declared that generative AI was not used in the creation of this manuscript.

Any alternative text (alt text) provided alongside figures in this article has been generated by Frontiers with the support of artificial intelligence and reasonable efforts have been made to ensure accuracy, including review by the authors wherever possible. If you identify any issues, please contact us.
